# Barriers to symptom management care pathway implementation in pediatric Cancer

**DOI:** 10.1186/s12913-021-07047-2

**Published:** 2021-10-09

**Authors:** L. Lee Dupuis, Allison Grimes, Emily Vettese, Lisa M. Klesges, Lillian Sung

**Affiliations:** 1 Program in Child Health Evaluative Sciences, The Hospital for Sick Children, Peter Gilgan Centre for Research and Learning, 686 Bay Street, Toronto, Ontario M5G 0A4 Canada; 2 Department of Pharmacy, The Hospital for Sick Children, 555 University Avenue, Toronto, Ontario M5G 1X8 Canada; 3 Leslie Dan Faculty of Pharmacy, University of Toronto, Toronto, Ontario M5S 3M2 Canada; 4 Pediatric Hematology Oncology, University of Texas Health Science Center at San Antonio, 7703 Floyd Curl Drive, San Antonio, TX 78229 USA; 5 Division of Public Health Sciences, Washington University School of Medicine, 4444 Forest Park Blvd, St. Louis, MO 63108 USA; 6 Division of Haematology/Oncology, The Hospital for Sick Children, 555 University Avenue, Toronto, Ontario M5G 1X8 Canada

**Keywords:** Pediatric oncology, Symptom management, Care pathway implementation, COVID-19 pandemic

## Abstract

**Background:**

Objectives were to describe barriers to pediatric cancer symptom management care pathway implementation and the impact of the COVID-19 pandemic on clinical research evaluating their implementation.

**Methods:**

We included 25 pediatric oncology hospitals in the United States that supported a grant submission to perform a cluster randomized trial in which the intervention encompassed care pathways for symptom management. A survey was distributed to site principal investigators prior to randomization to measure contextual elements related to care pathway implementation. Questions included the inner setting measures of the Consolidated Framework for Implementation Research (CFIR), study-specific potential barriers and the impact of the COVID-19 pandemic on clinical research. The Wilcoxon rank sum test was used to compare characteristics of institutions that agreed that their department supported the implementation of symptom management care pathways vs. institutions that did not agree.

**Results:**

Of the 25 sites, one withdrew because of resource constraints and one did not respond, leaving 23 institutions. Among the seven CFIR constructs, the least supported was implementation climate; 57% agreed there was support, 39% agreed there was recognition and 39% agreed there was prioritization for symptom management care pathway implementation at their institution. Most common barriers were lack of person-time to create care pathways and champion their use (35%), lack of interest from physicians (30%) and lack of information technology resources (26%). Most sites reported no negative impact of the COVID-19 pandemic across research activities. Sites with fewer pediatric cancer patients were more likely to agree that staff are supported to implement symptom management care pathways (*P* = 0.003).

**Conclusions:**

The most commonly reported barriers to implementation were lack of support, recognition and prioritization. The COVID-19 pandemic may not be a major barrier to clinical research activities in pediatric oncology.

**Supplementary Information:**

The online version contains supplementary material available at 10.1186/s12913-021-07047-2.

## Background

Most pediatric patients receiving cancer treatments experience bothersome symptoms that are poorly documented and often not treated [1, 2]. Symptom control is important as there is a strong correlation between increasing symptom burden and inferior quality of life [1]. Improving symptom control is likely to require multiple approaches including systematic symptom screening, symptom feedback to healthcare professionals and adherence to symptom management clinical practice guidelines (CPGs) [3, 4]. To address symptom screening, we developed the Symptom Screening in Pediatrics Tool (SSPedi), which measures the degree of bother for 15 symptoms considered most important by patients [5–7]. We then developed Supportive care Prioritization, Assessment and Recommendations for Kids (SPARK), which is a web-based application that consists of a symptom screening component centered on SSPedi and a supportive care CPG component [8–10].

To test whether SPARK can improve symptom control and quality of life for pediatric patients with cancer, we were awarded operating grants from the National Institutes of Health (1R01CA251112) and the Canadian Institutes of Health Research (PJT 169165) to perform a cluster randomized trial of 20 institutions in the United States. This trial will randomize 10 sites to intervention and 10 sites to control (usual care) groups. The intervention will include prompts to complete self-reported symptom screening three times weekly for 8 weeks for newly diagnosed pediatric patients with cancer (both inpatients and outpatients), symptom feedback to the primary healthcare team and adaptation of care pathways for symptom management. The care pathways will be adapted by each intervention site from generic pathways we developed using a standardized process to identify relevant CPGs [11]. Thus, intervention sites will need to adapt and implement evidenced-based care pathways. Planning of the trial started concurrent with the coronavirus disease pandemic (COVID-19) [12, 13] thus adding potential barriers.

There were limited reports on barriers to implementation of evidence-based care in pediatric oncology. For the care pathways to be successfully incorporated into routine practice, understanding implementation barriers was essential. We hypothesized that there would be barriers relevant to the planned trial including care pathway implementation, and that understanding those barriers would facilitate development of strategies to improve implementation. Thus, we circulated a survey to sites that had committed to the trial at the grant submission stage from which the 20 participating sites would be chosen. Objectives were to describe barriers to pediatric cancer symptom management care pathway implementation and the impact of the COVID-19 pandemic on clinical research evaluating their implementation.

## Methods

### Sites

The study was approved by the Research Ethics Board of The Hospital for Sick Children and the clinical trial registration number was NCT04614662. The sites were 25 hospitals providing care for pediatric oncology patients in the United States that supported a grant submission to conduct a cluster randomized trial focused on improving symptom control in pediatric cancer patients. The institutions were chosen to reflect variation in pediatric vs. mixed adult and pediatric sites and based upon previous research collaborations.

### Purpose of survey, survey creation and survey distribution

One component of the intervention is the institution-specific adaptation of symptom management care pathways that are based upon CPGs. The purposes of the baseline survey were to facilitate site selection (20 of the 25 potential sites would be chosen), to measure baseline characteristics of participating sites and to anticipate barriers and facilitators to trial conduct and care pathway implementation that could influence implementation processes. The trial will enroll 444 patients at 20 sites to demonstrate a difference of 3 points in total SSPedi scores between intervention and control patients, assuming alpha 0.05, power at least 80%, intraclass correlation coefficient 0.021 and SSPedi score standard deviation 8.8.

CPGs are statements developed to facilitate healthcare-related decisions; they are the foundations for translating evidence to clinical practice [14]. Rigorously developed CPGs must include a systematic review of the literature and convene a panel that weighs the benefits and harms of different treatment options to arrive at recommendations [14–16]. Care pathways are tools that can improve CPG implementation. They can be defined as “structured multidisciplinary care plans which detail essential steps in the care of patients with a specific clinical problem.” [17].

The survey (Additional file 1) measured baseline characteristics including institution, patient and healthcare professional characteristics. In order to understand potential barriers to symptom management care pathway implementation, we used three approaches. First, we used the Consolidated Framework for Implementation Research (CFIR) [18, 19]. The CFIR is a conceptual framework that includes factors that may influence intervention implementation. We focused on the inner setting measures that include the following constructs: culture, culture stress, culture effort, implementation climate, learning climate, leadership engagement and available resources. Culture may be described as a stable attribute of an organization that reflects its norms and values [20]. Of these constructs, implementation climate was central to our aims and thought to be especially important to successful implementation given its specificity to the task. It has been identified as being important to influencing implementation in practice [18, 21, 22]. Each of the questions was rated on a 5-point Likert scale consisting of 1 = “strongly disagree”; 2 = “disagree”; 3 = “neutral”; 4 = “agree”; and 5 = “strongly agree”. We dichotomized those who agreed (score of 4 or 5) vs. those who were neutral or disagreed (score of 1, 2 or 3).

We also used a second set of questions that were specific to the proposed study and asked if the lack of the following were potential barriers to symptom management care pathway adaptation and implementation: person-time to create care pathways and champion their use; education and mentorship around care pathway use; hospital leadership support; interest from physicians; interest from allied health; information technology resources; and collaboration between different disciplines. These were rated on a 5-point Likert scale representing the degree to which they were a barrier: 1 = “not at all”; 2 = “a little”; 3 = “somewhat”; 4 = “a lot”; and 5 = “extreme”. We focused on any barrier defined as those that were somewhat, a lot or extreme barriers, and severe barriers defined as those that were a lot or extreme barriers. Third, given the timing of the COVID-19 pandemic related to full grant funding (R01 notice of award July 2020), we also asked about the impact of the pandemic across the spectrum of clinical research activities.

The primary outcomes were related to support, recognition and prioritization of symptom management care pathway implementation from the CFIR implementation climate construct. The survey was piloted internally prior to distribution. It was disseminated to the participating sites by email and completed in REDCap. The respondents were the site principal investigators; they could consult with other institutional personnel to facilitate survey completion. Reminders were sent weekly up to three times in the event of non-response.

### Statistics

Descriptive statistics were used to summarize baseline characteristics and potential barriers to symptom management care pathway implementation. We compared institutional characteristics of those that agreed that their department supports, recognizes and prioritizes the implementation of symptom management care pathways vs. those that did not agree using the Wilcoxon rank sum test. Analyses were performed using R studio version 3.6.1, The R Foundation for Statistical Computing.

## Results

Of the 25 sites who supported grant submission, one withdrew because of institutional resource constraints and one did not complete the survey, leaving 23 institutions included in the analysis. The survey was completed between August 5, 2020 and September 9, 2020. Table 1 describes institutional, patient and healthcare professional characteristics. Within all institutions, health care professionals create orders in the electronic health record for symptom prevention and management. The median number of new pediatric cancer patients diagnosed annually was 90 (interquartile range (IQR) 63 to 200). All institutions were described as not-for-profit. The median number of physician full time equivalents was 9 (IQR 5 to 13).

**Table 1:** Demographic characteristics of institutions (*N* = 23)

	Value
**Institution Characteristics**	
Pediatric vs. Mixed Adult and Pediatric (%)	16 (70%)
Not-for-Profit vs. For-Profit (%)	23 (100%)
**Patient Characteristics**	
Median Number Pediatric Cancer Patients Diagnosed Annually (IQR)	90 (63 to 200)
Median Insurance Type Percentage (IQR)	
Private	48 (33 to 55)
Public	50 (44 to 65)
No insurance	1 (0 to 5)
Median Male Percentage (IQR)	53 (50 to 56)
Median Race Percentage (IQR)	
American Indian or Alaskan native	0 (0 to 1)
Asian	5 (3 to 10)
Black or African American	10 (5 to 21)
Native Hawaiian or other pacific islander	0 (0 to 1)
White	70 (64 to 87)
Median Hispanic or Latino Ethnicity (IQR)	26 (11 to 40)
Median Language Spoken Percentage (IQR)	
English	80 (71 to 90)
Spanish	12 (7 to 20)
Other	2 (1 to 5)
**Healthcare Professional Characteristics**	
Median MD or DO Full Time Equivalents (IQR)	9 (5 to 13)
Median Nurse Practitioner Full Time Equivalents (IQR)	5 (2 to 10)
Median Physician Assistant Full Time Equivalents (IQR)	1 (0 to 1)
Median MD or DO Years in Practice (IQR)	11 (10 to 15)
Median Nurse Practitioner Years in Practice (IQR)	8 (4 to 10)
Median Physician Assistant Years in Practice (IQR)	3 (0 to 10)

Abbreviations: MD, medical doctor; DO, doctor of osteopathy; IQR, interquartile range

Table 2 shows the results of the inner setting measures from the CFIR. Across most constructs, at least 70% of institutions agreed that the culture, climate, leadership and resources facilitated symptom management care pathway implementation. With respect to the implementation climate construct, less than 70% of respondents agreed or strongly agreed with the following positively framed statements: “department staff gets the support they need to implement care pathways for symptom management” (57%), “department staff gets recognition for implementing care pathways for symptom management” (39%) and “implementing care pathways for symptom management is a top priority of the department” (61%). Respondents from 35% of institutions indicated that “staff members often showed signs of stress and strain”.

**Table 2:** Inner setting measures from the consolidated framework for implementation related to symptom management care pathways (*N* = 23)

	n*	%
**Culture**		
People at all levels openly talk about what is and isn’t working	20	87%
Most people in this department are willing to change how they do things in response to feedback from others	16	70%
It is hard to get things to change in our department	4	17%
I can rely on the other people in this department to do their jobs well	21	91%
Most of the people who work in our department seem to enjoy their work	20	87%
Difficult problems are solved through face-to-face discussions	19	83%
We regularly take time to reflect on how we do things	16	70%
After trying something new, we take time to think about how it worked	17	74%
People in this department operate as a real team	20	87%
**Culture Stress**		
I am under too many pressures to do my job effectively	1	4%
Staff members often show signs of stress and strain	8	35%
The heavy workload here reduces program effectiveness	5	22%
Staff frustration is common here	5	22%
**Culture Effort**		
People in this department always want to perform to the best of their abilities	22	96%
People are enthusiastic about their work	21	91%
People in our department get by with doing as little as possible	0	0%
People are prepared to make a special effort to do a good job	20	87%
People in this department do not put more effort into their work than they have to	0	0%
**Implementation Climate**		
Department staff are expected to help the institution meet its goal	23	100%
Department staff gets the support they need to implement care pathways for symptom management	13	57%
Department staff gets recognition for implementing care pathways for symptom management	9	39%
Implementing care pathways for symptom management is a top priority of the department	14	61%
**Learning Climate**		
We regularly take time to consider ways to improve how we do things	20	87%
People in our department actively seek new ways to improve how we do things	22	96%
This department encourages everyone to share ideas	22	96%
This department learns from its mistakes	20	87%
When we experience a problem in the department, we make a serious effort to figure out what’s really going on	22	96%
**Leadership Engagement**		
The department leadership makes sure that we have the time and space necessary to discuss changes to improve care	19	83%
Leadership in this department creates an environment where things can be accomplished	19	83%
Department leadership promotes an environment that is an enjoyable place to work	18	78%
Leadership strongly supports department change efforts	21	91%
**Available Resources**		
In general, when there is agreement that change needs to happen in the department we have the necessary support in terms of: budget or financial resources	17	74%
In general, when there is agreement that change needs to happen in the department we have the necessary support in terms of: training	20	87%
In general, when there is agreement that change needs to happen in the department we have the necessary support in terms of: staffing	16	70%
The following are available to make implementing care pathways for symptom management work in our department: patient awareness/need	20	87%
The following are available to make implementing care pathways for symptom management work in our department: provider buy-in	19	83%
The following are available to make implementing care pathways for symptom management work in our department: intervention team	19	83%

* n - number of respondents who agreed or strongly agreed

Table 3 shows additional barriers to implementing symptom management care pathways among respondents. The most common barriers (somewhat, a lot or extreme barrier) were as follows: lack of person-time to create care pathways and champion their use (35%), lack of interest from physicians (30%) and lack of information technology resources (26%). Severe barriers (a lot or extreme barrier) were rare and the most common was the lack of person-time to create care pathways and champion their use (9%).

**Table 3:** Site-perceived barriers to developing and implementing care pathways (*N* = 23 Sites)

	n	%
Lack of person-time to create care pathways and champion their use		
Not at all or a little barrier	15	65%
Somewhat	6	26%
A lot or extreme barrier	2	9%
Lack of education and mentorship around care pathway use		
Not at all or a little barrier	18	78%
Somewhat	4	17%
A lot or extreme barrier	1	4%
Lack of hospital leadership support		
Not at all or a little barrier	18	78%
Somewhat	5	22%
A lot or extreme barrier	0	0%
Lack of interest from physicians		
Not at all or a little barrier	16	70%
Somewhat	7	30%
A lot or extreme barrier	0	0%
Lack of interest from allied health		
Not at all or a little barrier	21	91%
Somewhat	2	9%
A lot or extreme barrier	0	0%
Lack of information technology resources		
Not at all or a little barrier	17	74%
Somewhat	5	22%
A lot or extreme barrier	1	4%
Lack of collaboration between different disciplines		
Not at all or a little barrier	18	78%
Somewhat	4	17%
A lot or extreme barrier	1	4%

Table 4 illustrates the impact of the COVID-19 pandemic on research activities at the institutions. Most sites reported no negative impact of the pandemic across research activities. The most common activities that were a lot more difficult or almost impossible were executing contracts (9%), study activation (9%) and accessing patients in person (9%).

**Table 4:** Impact of COVID-19 pandemic on clinical research (*N* = 23)

	n	%
Obtaining institutional review board approval		
Better than usual or no impact	18	78%
A little more difficult	5	22%
A lot more difficult or almost impossible	0	0%
Executing contracts		
Better than usual or no impact	17	74%
A little more difficult	4	17%
A lot more difficult or almost impossible	2	9%
Study activation		
Better than usual or no impact	14	61%
A little more difficult	7	30%
A lot more difficult or almost impossible	2	9%
Accessing patients in person		
Better than usual or no impact	13	57%
A little more difficult	8	35%
A lot more difficult or almost impossible	2	9%
Accessing patients remotely in hospital		
Better than usual or no impact	21	91%
A little more difficult	2	9%
A lot more difficult or almost impossible	0	0%
Accessing patients remotely at home		
Better than usual or no impact	19	83%
A little more difficult	4	17%
A lot more difficult or almost impossible	0	0%
Accessing hospital systems		
Better than usual or no impact	19	83%
A little more difficult	4	17%
A lot more difficult or almost impossible	0	0%
Clinical research associate availability		
Better than usual or no impact	14	61%
A little more difficult	9	39%
A lot more difficult or almost impossible	0	0%

Table 5 compares the attributes of sites who agreed that symptom management care pathway implementation was supported, recognized and prioritized vs. those who did not agree with these statements. Sites with fewer newly diagnosed cancer patients and those with fewer physician, nurse practitioner and physician assistant full time equivalents were significantly more likely to agree that their staff are supported. Sites with a larger percentage of black patients were significantly more likely to agree that their staff receives recognition for implementing symptom management care pathways and that implementation is a priority.

**Table 5:** Support, recognition and priority of care pathway implementation by patient and healthcare professional characteristics

	Agree^**a**^	Neutral or Disagree	***P*** Value
**Department staff gets the support they need to implement care pathways for symptom management**	***N*** **= 13**	***N*** **= 10**	
Pediatric vs. Mixed Adult and Pediatric (%)	9 (69%)	7 (70%)	1.000
Median Number Pediatric Cancer Patients Diagnosed Annually (IQR)	66 (50 to 90)	200 (105 to 302)	0.003
Median Insurance Type Percentage (IQR)			
Private	38 (30 to 50)	51 (43 to 57)	0.202
Public	59 (50 to 65)	46 (42 to 53)	0.225
No insurance	0 (0 to 5)	2 (1 to 5)	0.276
Median Male Percentage (IQR)	55 (50 to 60)	52 (50 to 54)	0.281
Median Race Percentage (IQR)			
American Indian or Alaskan native	0 (0 to 2)	1 (0 to 1)	0.921
Asian	5 (1 to 10)	5 (3 to 9)	0.573
Black or African American	20 (8 to 23)	9 (5 to 17)	0.351
Native Hawaiian or other pacific islander	0 (0 to 1)	1 (0 to 1)	0.297
White	70 (55 to 89)	72 (70 to 80)	1.000
Median Hispanic or Latino Ethnicity (IQR)	20 (8 to 34)	32 (25 to 42)	0.291
Median Language Spoken Percentage (IQR)			
English	80 (72 to 91)	80 (71 to 88)	0.852
Spanish	12 (3 to 20)	14 (10 to 22)	0.534
Median MD or DO Full Time Equivalents (IQR)	7 (5 to 10)	13 (9 to 21)	0.014
Median Nurse Practitioner Full Time Equivalents (IQR)	2 (1 to 5)	10 (7 to 11)	0.009
Median Physician Assistant Full Time Equivalents (IQR)	0 (0 to 1)	1 (0 to 2)	0.033
Median MD or DO Years in Practice (IQR)	11 (10 to 15)	11 (10 to 15)	0.569
Median Nurse Practitioner Years in Practice (IQR)	5 (2 to 8)	10 (7 to 12)	0.053
Median Physician Assistant Years in Practice (IQR)	0 (0 to 10)	5 (1 to 9)	0.448
**Department staff gets recognition for implementing care pathways for symptom management**	***N*** **= 9**	***N*** **= 14**	
Pediatric vs. Mixed Adult and Pediatric (%)	7 (78%)	9 (64%)	0.824
Median Number Pediatric Cancer Patients Diagnosed Annually (IQR)	85 (60 to 110)	105 (67 to 200)	0.636
Median Insurance Type Percentage (IQR)			
Private	41 (30 to 60)	50 (36 to 52)	0.850
Public	59 (40 to 65)	48 (45 to 60)	0.752
No insurance	0 (0 to 2)	2 (0 to 5)	0.204
Median Male Percentage (IQR)	55 (50 to 57)	52 (50 to 55)	0.723
Median Race Percentage (IQR)			
American Indian or Alaskan native	0 (0 to 2)	1 (0 to 1)	0.840
Asian	5 (5 to 10)	4 (1 to 7)	0.098
Black or African American	20 (10 to 29)	8 (5 to 17)	0.037
Native Hawaiian or other pacific islander	1 (0 to 2)	0 (0 to 1)	0.245
White	68 (54 to 70)	75 (70 to 90)	0.037
Median Hispanic or Latino Ethnicity (IQR)	20 (8 to 30)	32 (18 to 58)	0.088
Median Language Spoken Percentage (IQR)			
English	80 (75 to 87)	80 (71 to 92)	0.825
Spanish	12 (8 to18)	14 (6 to 22)	0.570
Median MD or DO Full Time Equivalents (IQR)	8 (5 to 10)	11 (5 to 16)	0.256
Median Nurse Practitioner Full Time Equivalents (IQR)	2 (2 to 10)	6 (1 to 10)	0.898
Median Physician Assistant Full Time Equivalents (IQR)	0 (0 to 1)	1 (0 to 2)	0.058
Median MD or DO Years in Practice (IQR)	15 (10 to 20)	11 (10 to 14)	0.101
Median Nurse Practitioner Years in Practice (IQR)	5 (3 to 8)	10 (5 to 14)	0.100
Median Physician Assistant Years in Practice (IQR)	0 (0 to 3)	5 (0 to 10)	0.255
**Implementing care pathways for symptom management is a top priority of the department**	***N*** **= 14**	***N*** **= 9**	
Pediatric vs. Mixed Adult and Pediatric (%)	9 (64%)	7 (78%)	0.824
Median Number Pediatric Cancer Patients Diagnosed Annually (IQR)	83 (62 to 108)	200 (70 to 329)	0.122
Median Insurance Type Percentage (IQR)			
Private	41 (35 to 52)	50 (24 to 59)	0.658
Public	53 (45 to 64)	46 (41 to 75)	0.658
No insurance	2 (0 to 5)	1 (0 to 3)	0.494
Median Male Percentage (IQR)	53 (50 to 59)	53 (50 to 55)	0.822
Median Race Percentage (IQR)			
American Indian or Alaskan native	0 (0 to 1)	1 (0 to 1)	0.227
Asian	5 (3 to 10)	4 (3 to 8)	0.567
Black or African American	20 (9 to 25)	5 (4 to 10)	0.013
Native Hawaiian or other pacific islander	0 (0 to 0)	1 (0 to 1)	0.040
White	70 (58 to 83)	75 (70 to 88)	0.526
Median Hispanic or Latino Ethnicity (IQR)	28 (14 to 40)	26 (5 to 34)	0.636
Median Language Spoken Percentage (IQR)			
English	78 (71 to 90)	85 (75 to 89)	0.614
Spanish	15 (9 to 20)	10 (3 to 20)	0.591
Median MD or DO Full Time Equivalents (IQR)	9 (6 to 10)	12 (5 to 16)	0.449
Median Nurse Practitioner Full Time Equivalents (IQR)	3 (2 to 7)	9 (1 to 11)	0.292
Median Physician Assistant Full Time Equivalents (IQR)	0 (0 to 1)	1 (0 to 2)	0.222
Median MD or DO Years in Practice (IQR)	11 (10 to 15)	12 (10 to 15)	0.974
Median Nurse Practitioner Years in Practice (IQR)	8 (4 to 14)	7 (4 to 10)	0.704
Median Physician Assistant Years in Practice (IQR)	2 (0 to 10)	5 (0 to 5)	0.893

*Abbreviations*: *IQR* interquartile range, *MD* medical doctor, *DO* doctor of osteopathy

^a^ Rated on a 5-point Likert scale ranging from strongly disagree to strongly agree. Those who stated they agreed or strongly agreed were categorized as “agree”

## Discussion

In this study, we found that few survey respondents anticipated challenges with care pathway implementation. However, potential barriers that were reported lay within the implementation climate construct of CFIR and were lack of support, recognition and prioritization at the participating institutions. Lack of person-time to create care pathways and champion their use, lack of physician interest, and lack of information technology were other important potential barriers reported with respect to developing and implementing symptom management care pathways. However, the COVID-19 pandemic did not appear to be perceived as a major barrier to research conduct.

We found that unfavorable implementation climate may be a potential barrier to care pathway implementation. Other studies have also identified this construct as a potential problem in program implementation [23, 24]. It is interesting that smaller sites reported receiving more support for care pathway implementation. It is possible that smaller sites are more likely to provide verbal and non-verbal support of initiatives in general and if this is true, identifying ways to provide this type of support across institutions could be important. Such support could include wide availability of research staff to address questions and provide educational materials. It may also be important to identify site champions who can provide this type of support locally. More specifically, our proposed strategy will focus on enhancing support and recognition for care pathway implementation although these elements will primarily arise extrinsic to the institution and be provided by the study team.

The finding of greater support at institutions with a greater percentage of black patients may be spurious. It also may reflect additional supports given to institutions that are minority based, such as those participating in the National Cancer Institute Community Oncology Research Program (a program aimed at sites with greater representation of racial/ethnic minorities or rural residents). We also found that lack of physician interest was a potential barrier to developing and implementing symptom management care pathways. Future qualitative research could explore reasons behind lack of interest and identify potential interventions to mitigate or address the issue.

We found that the COVID-19 pandemic was not perceived as a major barrier to research conduct. This finding is in contrast to a recent meta-analysis suggesting that trial delays and cessation were common and were a direct consequence of the pandemic [25]. Our findings may differ because the survey was distributed later in the pandemic, when many institutions had adapted to it. Further, some institutions have made distinctions based upon whether research activities are in person vs. not in person and whether they are essential vs. not essential. Given that our trial could be conducted entirely remotely, and since some could consider this type of trial essential, respondents may have anticipated fewer barriers compared with other research studies. Lastly, respondents to our survey may not have had personal experience surmounting the logistical and bureaucratic barriers to the conduct of research created by the COVID-19 pandemic and, thus, may have not fully appreciated its impact. While our findings are unique to this particular project, they offer useful insight into institutional support of research during the pandemic.

The strengths of this study include the utilization of an established framework (CFIR) in addition to study-specific items in order to identify potential barriers to care pathway implementation. This will allow important contextual elements to be evaluated for their future relationship to implementation strategies and outcomes. Another strength is the evaluation of the impact of the COVID-19 pandemic on clinical research, a timely and important question. However, the study is limited as two institutions either dropped out or did not complete the survey; they are likely to be different than the 23 institutions that did complete the survey. In addition, these questions were mainly answered by a single individual, namely the site principal investigator. While input from other colleagues could have been accessed, perspectives of other healthcare professionals at the site were not fully represented. Also, sites agreed to provide support at the grant submission stage, suggesting they believe that symptom management is important. Thus, participating sites are likely a positively biased cohort and the “typical” site may report more barriers to care pathway implementation. Finally, evaluation of characteristics of sites who agreed that their department supports, recognizes and prioritizes the implementation of symptom management care pathways vs. those who did not agree should be considered hypothesis generating.

## Conclusions

In conclusion, respondents at pediatric oncology institutions expected few barriers to symptom management care pathway implementation at their institutions. The most commonly reported barriers to implementation were lack of support, recognition and prioritization. The COVID-19 pandemic may not be a major barrier to clinical research activities in pediatric oncology. These results were limited by being conducted at sites that have prioritized supportive care. Future work should use this information to improve care pathway implementation.

## Supplementary Information

**Additional file 1.**

## Data Availability

The datasets used or analyzed during the current study are available from the corresponding author on reasonable request.

## Abbreviations

CFIR: Consolidated Framework for Implementation Research
CPG: Clinical Practice Guideline
SSPedi: Symptom Screening in Pediatrics Tool
SPARK: Supportive care Prioritization, Assessment and Recommendations for Kids
COVID-19: Coronavirus disease pandemic
