# Supplementary material for: Barriers to symptom management care pathway implementation in pediatric Cancer
Source: BMC Health Serv Res. 2021 Oct 9;21:1068. doi: 10.1186/s12913-021-07047-2 (PMC8500815; doi:10.1186/s12913-021-07047-2)
Supplement: Supplementary file 1 — Additional file 1. [file 12913_2021_7047_MOESM1_ESM.docx]

**Site Name:**

**Institutional Information Related to Clinical Practice Guidelines and Care Pathways for Symptoms**

*This section relates to approaches to prevent and treat* ***symptoms*** *such as fatigue, pain, constipation and nausea. It is NOT related to management of fever and neutropenia, prevention of bacterial or fungal infections or VTE management as examples (i.e. non-symptom toxicities).*

1. Is it your institutional standard of care to formally screen pediatric cancer patients in order to identify more than one symptom?

Yes

No

We are interested in clinical practice guidelines (CPGs) and care pathways. They are compared and contrasted in the Table below.

|  | **Clinical Practice Guideline** | **Care Pathway** |
| --- | --- | --- |
| Content | Set of recommendations – often a peer-reviewed publication | Instructions or orders - usually not a publication and often developed by each institution |
| Specificity | Broad such as: use exercise to manage fatigue | Specific such as: consult physiotherapy if severe fatigue |
| Institutional Choices | Weak recommendations are shown, specifying that institutions may choose to implement or not implement | Either the recommendation will be included or excluded depending on center choice |
| Format | Document | May be a document or order set (including pre-printed orders) |

1. Does your institution use CPGs for the prevention and management of symptoms?

Yes

No (go to question 5)

1. If yes, for which symptoms does your institution use CPGs? (check all that apply)

Fatigue  Nausea/vomiting

Pain  Anorexia

Constipation  Other, specify:

1. If yes, to what extent are the CPGs routinely used by healthcare professionals?

Never

A little

Somewhat

A lot

Always

1. Does your institution use care pathways for the prevention and management of symptoms?

☐ Yes

☐ No (go to question 9)

1. If yes, for which symptoms does your institution use care pathways? (check all that apply)

☐ Fatigue ☐ Nausea/vomiting

☐ Pain ☐ Anorexia

☐ Constipation ☐ Other, specify:

1. If yes, in general, to what extent are the institutional care pathways routinely used by healthcare professionals?

☐ Not at all

☐ A little

☐ Somewhat

☐ A lot

☐ Almost always

1. If yes, how do health care professionals at your institution access care pathways (i.e. know what interventions to use)? (check all that apply)

☐ Electronic health record through alerts

☐ Electronic health record through order sets

☐ Institutional app or website

☐ Shared hard drive

☐ Paper document (may be emailed but does not reside on an institutional website or shared drive)

☐ Other, describe:

1. In your institution, do health care professionals create orders within the electronic health record (i.e. computerized physician order entry)?

☐ Yes

☐ No (go to 11)

1. If yes, how easy or difficult is it for a new order set to be created in your electronic health record?

☐ Not difficult at all

☐ A little

☐ Somewhat

☐ A lot

☐ Extremely difficult

1. In your institution, does your electronic health record allow symptoms to be recorded in a structured format (i.e. similar to laboratory results)?

☐ Yes

☐ No

1. For institutions randomized to the intervention group, we will facilitate the development of institution-specific adapted care pathways for the management of 15 symptoms. To what extent do you think the **lack** of the following will be barriers to developing care pathways and having them be used at your institution?

|  | Not at all a barrier | A little | Somewhat | A lot | Extreme barrier |
| --- | --- | --- | --- | --- | --- |
| Lack of person-time to create care pathways and champion their use |  |  |  |  |  |
| Lack of education and mentorship around care pathway use |  |  |  |  |  |
| Lack of hospital leadership support |  |  |  |  |  |
| Lack of interest from physicians |  |  |  |  |  |
| Lack of interest from allied health |  |  |  |  |  |
| Lack of information technology (IT) resources |  |  |  |  |  |
| Lack of collaboration between different disciplines |  |  |  |  |  |

**Institutional Demographics**

1. Is your institution a pediatric or mixed pediatric/adult site?

☐ Pediatric

☐ Mixed Site

1. Is your institution for-profit or not-for-profit?

☐ For-Profit

☐ Not-For-Profit

1. How many pediatric (0-18 years) patients are newly diagnosed with cancer annually at your site?
2. How many patients are newly diagnosed with cancer, are 8-18 years old at diagnosis, and speak English, Spanish or French annually at your site?

**The following questions only refer to the pediatric (0-18 years) cancer population at your institution**

1. Patient payment type (%):

% Private (includes employer-based and ACA plans)
% Public (state/federal)

% No insurance

1. Patient sex (%):

% Male

% Female

1. Patient race (%):

% American Indian or Alaska Native

% Asian

% Black or African American

% Native Hawaiian or Other Pacific Islander

% White

% Other

1. Patient ethnicity (%):

% Non-Hispanic

% Hispanic or Latino

1. What is the primary language (language spoken at home) of pediatric cancer patients at your site?

% English

% Spanish

% French

% Other

# The following questions refer to providers at your institution

1. What are the total provider FTEs who care for pediatric oncology patients?
   MD/DO

Nurse practitioner

Physician assistant

1. What is the median provider years in practice working with pediatric oncology patients?

MD/DO

Nurse practitioner

Physician assistant

**Institutional Information Related to COVID-19**

1. Has there been any impact on clinical research due to the COVID-19 pandemic?

☐ Yes

☐ No (go to CFIR)

1. If yes, describe the impact of COVID-19 on clinical research in the following areas?

|  | Better than usual | No impact | A little more difficult | A lot more difficult | Almost impossible |
| --- | --- | --- | --- | --- | --- |
| Obtaining IRB approval |  |  |  |  |  |
| Executing contracts |  |  |  |  |  |
| Study activation |  |  |  |  |  |
| Accessing patients in person |  |  |  |  |  |
| Accessing patients remotely in hospital |  |  |  |  |  |
| Accessing patients remotely at home |  |  |  |  |  |
| Accessing hospital systems |  |  |  |  |  |
| CRA availability |  |  |  |  |  |

1. How is supportive care clinical research currently allowed to be conducted at your institution?

☐ In person only

☐ Remote only

☐ Both in person and remote

1. Does your institution allow virtual consent (eConsent)?

☐Yes
☐No

1. Are there any other limitations at your site due to COVID-19 that may impact this study?

**Inner Setting Measures from the Consolidated Framework for Implementation Research**

Instructions: The questionnaire consists of scales that measure constructs within the *Inner Setting* domain of the Consolidated Framework for Implementation Research (CFIR). Please indicate your level of agreement for each item.

| Culture | Strongly Disagree  1 | Disagree 2 | Neutral  3 | Agree 4 | Strongly Agree 5 |
| --- | --- | --- | --- | --- | --- |
| 1. People at all levels openly talk about what is and isn’t working |  |  |  |  |  |
| 2. Most people in this department are willing to change how they do things in response to feedback from others |  |  |  |  |  |
| 3. It is hard to get things to change in our department |  |  |  |  |  |
| 4. I can rely on the other people in this department to do their jobs well |  |  |  |  |  |
| 5. Most of the people who work in our department seem to enjoy their work |  |  |  |  |  |
| 6. Difficult problems are solved through face-to-face discussions |  |  |  |  |  |
| 7. We regularly take time to reflect on how we do things |  |  |  |  |  |
| 8. After trying something new, we take time to think about how it worked |  |  |  |  |  |
| 9. People in this department operate as a real team |  |  |  |  |  |

| Culture Stress | Strongly Disagree  1 | Disagree 2 | Neutral  3 | Agree 4 | Strongly Agree 5 |
| --- | --- | --- | --- | --- | --- |
| 1. I am under too many pressures to do my job effectively |  |  |  |  |  |
| 2. Staff members often show signs of stress and strain |  |  |  |  |  |
| 3. The heavy workload here reduces program effectiveness |  |  |  |  |  |
| 4. Staff frustration is common here |  |  |  |  |  |

| Culture Effort | Strongly Disagree  1 | Disagree 2 | Neutral  3 | Agree 4 | Strongly Agree 5 |
| --- | --- | --- | --- | --- | --- |
| 1. People in this department always want to perform to the best of their abilities |  |  |  |  |  |
| 2. People are enthusiastic about their work |  |  |  |  |  |
| 3. People in our department get by with doing as little as possible |  |  |  |  |  |
| 4. People are prepared to make a special effort to do a good job |  |  |  |  |  |
| 5. People in this department do not put more effort into their work than they have to |  |  |  |  |  |

| Implementation Climate | Strongly Disagree  1 | Disagree 2 | Neutral  3 | Agree 4 | Strongly Agree 5 |
| --- | --- | --- | --- | --- | --- |
| 1. Department staff are expected to help the institution meet its goal (i.e., improve symptom control for patients) |  |  |  |  |  |
| 2. Department staff gets the support they need to implement care pathways for symptom management |  |  |  |  |  |
| 3. Department staff gets recognition for implementing care pathways for symptom management |  |  |  |  |  |
| 4. Implementing care pathways for symptom management is a top priority of the department |  |  |  |  |  |

| Learning Climate | Strongly Disagree  1 | Disagree 2 | Neutral  3 | Agree 4 | Strongly Agree 5 |
| --- | --- | --- | --- | --- | --- |
| 1. We regularly take time to consider ways to improve how we do things |  |  |  |  |  |
| 2. People in our department actively seek new ways to improve how we do things |  |  |  |  |  |
| 3. This department encourages everyone to share ideas |  |  |  |  |  |
| 4. This department learns from its mistakes |  |  |  |  |  |
| 5. When we experience a problem in the department, we make a serious effort to figure out what’s really going on |  |  |  |  |  |

| Leadership Engagement | Strongly Disagree  1 | Disagree 2 | Neutral  3 | Agree 4 | Strongly Agree 5 |
| --- | --- | --- | --- | --- | --- |
| 1. The department leadership makes sure that we have the time and space necessary to discuss changes to improve care |  |  |  |  |  |
| 2. Leadership in this department creates an environment where things can be accomplished |  |  |  |  |  |
| 3. Department leadership promotes an environment that is an enjoyable place to work |  |  |  |  |  |
| 4. Leadership strongly supports department change efforts |  |  |  |  |  |

| Available Resources | Strongly Disagree  1 | Disagree 2 | Neutral  3 | Agree 4 | Strongly Agree 5 |
| --- | --- | --- | --- | --- | --- |
| 1. In general, when there is agreement that change needs to happen in the department we have the necessary support in terms of: budget or financial resources |  |  |  |  |  |
| 2. In general, when there is agreement that change needs to happen in the department we have the necessary support in terms of: training |  |  |  |  |  |
| 3. In general, when there is agreement that change needs to happen in the department we have the necessary support in terms of: staffing |  |  |  |  |  |
| 4. The following are available to make implementing care pathways for symptom management work in our department: patient awareness/need |  |  |  |  |  |
| 5. The following are available to make implementing care pathways for symptom management work in our department: provider buy-in |  |  |  |  |  |
| 6. The following are available to make implementing care pathways for symptom management work in our department: intervention team |  |  |  |  |  |

Citation: Fernandez ME, Walker TJ, Weiner BJ, et al. Developing measures to assess constructs from the Inner Seeting domain of the Consolidated Framework for Implementation Research. *Implementation Science.* 2018;13(1):52

A Cancer Prevention and Control Research Network (CPCRN) collaboration.

For questions about the measures or to reproduce, distribute, or display, please obtain permission by contacting Maria Fernandez ([Maria.E.Fernandez@ut.tmc.edu](mailto:Maria.E.Fernandez@ut.tmc.edu)) and Tim Walker (timothy.j.walker@uth.tmc.edu).
